# Covid-19: teaching and learning in practical courses under special regulations – a qualitative study of dental students and teachers

**DOI:** 10.1186/s12909-022-03656-5

**Published:** 2022-08-03

**Authors:** Katrin Hertrampf, Hans-Jürgen Wenz, Katja Goetz

**Affiliations:** 1 Department of Oral and Maxillofacial Surgery, University Hospital Schleswig-Holstein, Arnold-Heller Str. 3, Building B, Campus Kiel, 24105 Kiel, Germany; 2 Department of Prosthodontics, Propaedeutics and Dental Materials, University Hospital Schleswig-Holstein, Campus Kiel, Kiel, Germany; 3 Institute of Family Medicine, University Hospital Schleswig-Holstein, Campus Lübeck, Lübeck, Germany

**Keywords:** Covid-19, Dental education, Practical course, Qualitative study, Special regulations

## Abstract

**Background:**

The coronavirus pandemic led to a lockdown of public life. For universities, this meant suspensions or corresponding adaptations of practical courses. In Germany, Kiel Dental Clinic received special permission to start practical courses under appropriate hygiene conditions. The study aimed at recording the experiences and associated challenges of course implementation under the special regulations from the perspective of students and teachers.

**Methods:**

Qualitative guided interviews were conducted with students and teachers at Kiel in the summer semester 2020. Students (4th, 6th, 8th, 10th semesters) were recruited and lecturers responsible for conducting the practical courses within the dental clinic’s four departments. Evaluation was carried out by means of qualitative content analysis, whereby deductive procedures were supplemented by inductive ones.

**Results:**

Thirty-nine students and 19 lecturers took part. The flow of information at the start of the course was welcomed by students and teachers across the board. The lack of or limited adjustment to the scope tended to be assessed positively by students. The majority of both groups suspected there had been no reduction in learning, and learning had been improved due to the smaller group sizes. Regarding the necessary conditions for conducting the course, positive and negative aspects became apparent.

**Conclusion:**

Students and teachers felt very relief to start the practical courses under special conditions although the implementation was very challenging for both groups. The structural and content-related course adaptations required a high degree of flexibility on the part of students and lecturers alike, but also meant that courses were able to be conducted without serious deficits in learning gains.

**Supplementary Information:**

The online version contains supplementary material available at 10.1186/s12909-022-03656-5.

## Introduction

In December 2019, an outbreak of a new coronavirus was reported in Wuhan, China. Following China, a number of European countries subsequently reported increasing cases. At the end of January, the WHO declared the situation to be a Public Health Emergency of International Concern [1, 2]. Since then, this viral infection has developed into a global pandemic with 284.683.062 positive cases and 5.424.970 deaths reported worldwide [3].

Due to the virus’s global spread, Germany, like many other countries, decided to implement a national lockdown from March 2020. One of the measures for reducing the number of infections was the introduction of social distancing regulations in public and private life [4]. As a result, universities worldwide decided to suspend face-to-face teaching for the summer semester and to take learning digital [5–7]. Restrictions were first relaxed from mid-May.

In the case of dentistry, this led to a discussion in the university sector as to how digital teaching might look for a very practical study programme such as this, since dental education cannot be taught digitally; this is especially the case when it comes to very early and ongoing patient contact within clinical treatment – aspects which are considered essential to the dental curriculum [8–10]. Furthermore, students would lose competencies in routine treatment processes [11].

Against the backdrop of the relatively low incidence rates in Germany’s northernmost federal state, senior staff at the Kiel Dental Clinic developed a comprehensive social distancing and hygiene concept for the practical courses and liaised with the relevant local health authority, university, and university hospital. On the basis of this concept, the dental clinic was the first clinic in Germany to receive approval under a special provision to conduct in-person practical courses with patients from the beginning of May 2020. This regional decision was made possible due to Germany’s federal system and each university can make their own decision independent from the national government. This special reguirements are, in contrast for example to countries such as the USA, where a national decision led to the suspension of in-person teaching throughout the country [6, 12]. Implementation of this comprehensive social distancing and hygiene concept presented students and lecturers with significant challenges. Nevertheless, the way students and lecturers experienced this approach to conducting a practical dental course under special provisions had not yet been sufficiently investigated, as no one had yet experienced a pandemic with such drastic consequences [6].

The aim of the study was thus to explore the impact of the pandemic-related restrictions on the teaching of dentistry at Kiel in terms of assessments, experiences, obstacles and barriers from the perspective of dental students and their lecturers. The focus was on the teaching and learning situation in simulation and clinical treatment courses under the special regulations for face-to-face courses.

## Materials and methods

### Study design

The study was designed following a qualitative approach in the form of structured guided interviews exploring student and teaching staff experiences of the specific regulations governing practical dental courses during the Covid-19 pandemic. The COREQ checklist (Additional file 1) for comprehensive reporting of qualitative studies was used [13].

### Implementation of social distancing and hygiene guidelines

From the beginning of the summer semester, staff covering the theoretical subjects were asked to teach their sessions as live-streamed units or to make the semester’s teaching available to students digitally in a more compact format. This generated potential extra time for other course activities. However, no practical content was taught online. For the practical courses the special regulations included strict spacing and hygiene guidelines, affecting traffic flows within the building on the one hand and the flow of the individual practical courses on the other. A one-way system was set up within the building. In addition to the main entrance, specific doors were allocated for students entering and leaving the building. Required routes were laid out to each of the seminar rooms. Every student was given a fixed time to enter and leave the building. Group sizes were also significantly reduced for all courses. As a result, session start times were spread out over the day while students’ overall teaching hours were reduced. This required a partial introduction of shift work for staff. Simulation courses were thus allowed to commence from 4 May 2020. Clinical treatment courses followed from 11 May 2020.

### Recruitment

Students were informed about the study and recruited via video conference during courses in the last third of the summer semester. The lecturers from the various departments were given personal presentations on the project. Participation was voluntary for all. Interview appointments were subsequently arranged with the participants in person or by email. Data collection took place from June to August 2020. All interviews were conducted by two female members of the working group (KH, KG) either in person or by telephone. Both were experienced in performing qualitative research. As described in the literature, no difference in data quality was observed between face-to-face and telephone interviewing, and both may be recommended for use in the same qualitative study [14].

Both the course of the interviews themselves as well as their documentation followed the same predefined quality criteria. This included documentation of the time and of possible problems or interruptions encountered during the interview. Each participant received a short socio-demographic questionnaire before the interview began.

### Participants

Qualitative interviews were conducted with a purposive sample of students and teaching staff. Students from semesters four, six, eight, and ten at the dental school in Kiel, Germany were included in the study along with associated teaching staff. Dental simulation courses took place in the 4th and 6th semesters and clinical treatment courses in the 7th–10th semesters. The 8th and 10th semesters were selected as examples for the treatment courses. The target sample consisted of students from these semesters (*n* = 10 each) and lecturers from the four clinics, the pre-clinic, and the departmental directors (*n* = 19 in total), taking into account theoretical saturation [15].

The inclusion criteria for the students were as follows: membership of the respective subject semesters, over 18 years, and having sufficient knowledge of the German language. For the lecturers, inclusion criteria were responsibility for teaching content and its delivery in one of the Dental Clinic’s four departments, over 18 years, and having sufficient knowledge of the German language.

### Data collection

A semi-structured interview guide was developed by an interdisciplinary team comprising a sociologist, health services researcher, physician, and dental practitioners. Following a literature review and discussion within the study team, the interview guide focussed on two main themes:

- Implementation of specific regulations within the practical dental courses,
- Adaptation of the course structure due to specific regulations.

The interview guide (Additional file 2) was identical for both students and teaching staff and was tested with a student and lecturer for comprehensibility and sequencing of the individual questions. No changes were required. The test interviews were not included in the final analysis.

### Data analysis

All interviews were digitally audio recorded and transcribed in full verbatim. Transcripts were not submitted to participants for comments or correction. The texts were anonymised during transcription before undergoing qualitative content analysis [16]. The ATLAS.ti 8.4 (Scientific Software Development GmbH, 2020) software was used to assist with data analysis. The research team used a deductive-inductive approach to generating thematic categories. Firstly, a provisional category system was developed deductively based on the interview guidelines. The provisional category system was then adjusted during analysis according to the content of the transcripts. Any new categories which emerged were then added following an inductive approach. Transcripts were coded independently into main and sub-categories by two researchers (KH [dental practitioner background] and KG [health services research background]), following intensive discussions which continued until consensus was achieved. No inter-coder agreement was calculated. Saturation was reached when during the analyzing process nothing new data were added. The authors orientated on the concept of theoretical and data saturation [15]. Participant quotations were translated from German into English for publication purposes.

### Ethical approval

The project was approved by the Ethics Committee of the University Kiel, Germany (D509/20) and was conducted in accordance with the Declaration of Helsinki. Informed consent was obtained via a signed consent form and included permission for publication of anonymised quotes.

## Results

### Sample characteristics

Fifty-eight interviews were performed in total, divided between 39 with dental students and 19 with teaching staff. Nobody refused his or her participation. Interview duration varied and was 31.2 minutes on average for the dental student group (min. = 22 minutes, max. = 50 minutes) and 30.8 minutes for the teaching staff (min. = 15 minutes, max. = 41 minutes). Participant characteristics are shown in Table 1.

**Table 1:** Description of the study population

Variable	Students (***N*** = 39)	Lecturers (***N*** = 19)
**Women**	27	6
**Men**	12	13
**Age (mean)**	25.2	44.0
**(Age range)**	(20–31)	(31–65)
**Apprenticeship**	13	–
**Further completed study**	5	–
**Additional qualification**	5	–
**Departmental director**	–	4
**Course instructor**	–	7
**Course assistant**	–	8

Two main themes “implementation of specific regulations in the practical dental courses” and “adaptation of the course structure due to specific regulations” are presented in the following sections. Quotations are used to illustrate relevant aspects reported by participating students (S) and teaching staff (TS). An overview of all main categories and their definitions is given in Table 2.

**Table 2:** Main categories and their definition

Main category	Definition
Discrepancy between performing and pragmatism	Feelings and reactions regarding the practical courses which began under specific regulations.
The emotional level: relief	Feelings of relief regarding the starting of practical courses.
Reflection on performance	Positive and negative experiences of the courses and of the whole semester.
Resignation	Feelings of resignation due to different circumstances concerning the implementation of the practical courses.
Uncertainty concerning risk of infection	Various concerns about infecting patients or students during the practical course as well as general concerns regarding the pandemic and infection risk.
Adaptation of the structure	Positive and negative feelings concerning the adaption of the structure of the practical courses.
Adaptation of the scope	Attitudes to the adaption of the scope.
Lack of free practice time	Feelings of unsecure due to the perceived lack of free practice time.
Questioning personal responsibility	The teaching staff perspective on students studying under this specific regulation.
Course learning gain	The range from less to more to the gain from the course.
Support from teachers	Described how students perceived support from the teaching staff.
Concern about low quality	Attitudes to concerns about lower quality due to the specific regulations in the practical courses.

### Theme: Implementation of specific regulations in the practical dental courses

**Fig. 1:**
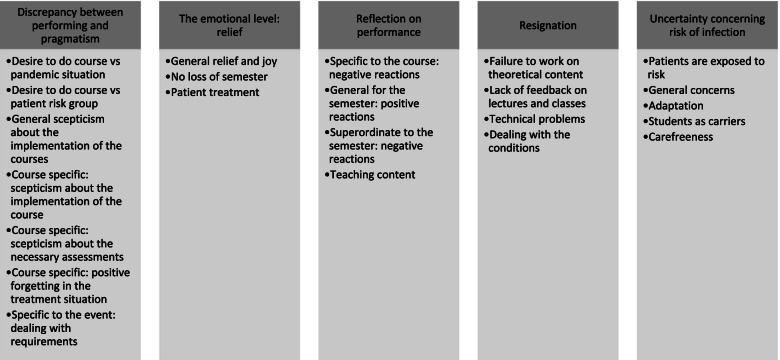
Implementation of special regulations in the practical dental courses - Main- and subcategories

This theme describes how students and teaching staff perceived the implementation of specific regulations on carrying out the practical dental courses with patients. For this theme, five main categories were formed and divided into the different sub-categories shown in Fig. 1.

#### Discrepancy between performance and pragmatism

The discrepancy between performance and pragmatism (main category) described student and teaching staff feelings and reactions regarding the practical dental courses which began under specific regulations. This category comprised various inner conflicts concerning the situation created by pandemic, concerning patient risk, and concerning the desire to get on with the course. One student described their inner conflict as follows: “And I was positive at the beginning because I thought, ‘This is it, no studying any more!’ On the other hand, I kept thinking, ‘How can I call older people with a clear conscience to get them to come to the clinic?’” (S21). Students and teaching staff reported a general scepticism regarding taking part in the practical course as a statement of a teaching staff illustrated: “Yeah, so before the whole thing started we already had concerns about whether the hygiene guidelines would work out that way, whether our students would really adhere to them in the end, whether our patients would come” (TS04). Concerns were also expressed regarding assessment as one student argued: “I hope we can still do it just as well, because the time was shortened, too. [...] And the same level of performance was still expected, of course” (S25). Moreover, the introduction of the specific regulations was perceived as a challenge not only for students and teaching staff but also for the curriculum as showed by the following quotations of a teaching staff: “And then all of a sudden there were conflicts with our seminar times because students couldn’t get home quickly enough to follow the seminar online” (TS06).

#### The emotional level: relief

The implementation of specific regulations for conducting practical dental courses with patients was associated with an emotional feeling of relief among both students and teaching staff. Alongside general relief, everyone was happy not to be losing a semester. One student expressed their feelings as follow: “When we were really allowed to come here, that was very positive, because then you had the hope that, OK, this is going to work. I don’t have to continue my studies for an extra half a year” (S21). Moreover, the students were relieved that patient treatment could continue as following statement showed: “The fact that we were able to continue treating patients, too, was a great relief – we were really happy” (S06).

#### Reflection on performance

The main category “reflection on performance” included positive and negative experiences of the courses and of the whole semester. Negative course experiences were a result of the specific regulations. One member of teaching staff stated: “But since we have this tight time frame and just have a certain amount of time when we have to get in and out of the building again, all our time has gone after the end of treatment and leaving the building” (TS16). Furthermore, teaching staff reflected very critically on their delivery of teaching material as demonstrated by the following statement: “The students expected more from us, I think; they expected a thread running through it all and a kind of precise specification [...] we just couldn’t always meet their expectations” (TS08).

#### Resignation

The implementation of specific regulations sometimes led to a kind of resignation. We identified this main category for the teaching staff only. “Resignation” comprises different obstacles such as a lack of feedback, technical problems, inadequate preparation of theoretical content and dealing with the special regulations. One member of teaching staff stated their frustration with the special regulations as follows: “It was stressful and with the ... it all starts with the admission planning for [...] bringing people into the building – I couldn’t imagine how it would work” (TS16).

#### Uncertainty concerning the risk of infection

A further main category, “uncertainty concerning the risk of infection”, described the various concerns about infecting patients or students during the practical course as well as general concerns regarding the pandemic and infection risk. Following statement from the perspective of teaching staff illustrated this concern: “That we might get infected and then have a total lockdown here. You don’t want that under any circumstances, of course. I was a little afraid of that happening” (TS10). Some students, however, described a carefree attitude, as shown by the following student statement: “I think I always felt that I was in good hands. I didn’t really have any major concerns for myself” (S11).

### Theme: Adaptation of the course structure due to specific regulations

**Fig. 2:**
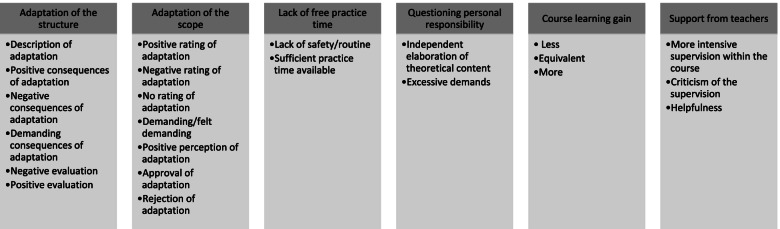
Adaption of the course structure due to specific regulations - Main- and subcategories

This theme describes how students and teaching staff perceived the adaptation of the course structure due to specific regulations. For this theme, six main categories were derived and divided into the different sub-categories shown in Fig. 2.

#### Adaptation of the structure

The “adaptation of the course structure” main category included different subcategories such as description of the adaptation, positive and negative consequences, and assessment of the adaptation. Students and teaching staff reported that the adaptation was dependent on aspects of hygiene rules and clear requirements for participation in the course. Moreover, it led to a higher workload for both groups. A member of the teaching staff stated this specific adaption as follow: “Yes, for the students – the regulations, admission rules and masks, social distancing and so on, they kept to them. Yes, so that worked well [...]” (TS14). In comparison, following statement of one student described the negative aspects of such specific regulations:

“So you prepared teeth or did the work for the same amount of time as in the normal semester; so you spent longer in the clinic on the day, so to speak” (S34).

The different hygiene rules such as staggered entrance times were evaluated as a positive consequence of the adaptation process as the statement of a teaching staff member showed: “So, some of the changes are good, I think, and I even think that if it’s feasible in terms of staffing, you should keep some of the changes. So this staggered start is relatively pleasant compared to starting a whole group together” (TS09). For students in particular, the loss of patient debriefing with the whole semester and the dentist was a negative consequence of the adaptation of the course structure. However, in general, students and teaching staff evaluated the adaptation positively. Teaching staff felt that they could more freely structure their course: “The freedom of design, the removal of all the boundaries that were previously set in stone – it was brilliant! You could really try out things that you wanted to do” (TS13). Students evaluated the reduction in person numbers per course especially positively: “I actually thought the course implementation was really good. The fact that the groups were made smaller, too” (S20).

#### Adaptation of the scope

There was little to no adaptation of the scope. Most of the students and teaching staff assessed this lack of adaptation as a positive. One student stated: “I think it’s good in general, actually, because I think that you should learn something during your studies, too” (S06). One teaching staff member illustrated: “So they didn’t kind of turn a blind eye here” (TS11). However, some students thought that the lack of adaptation led to more stress: “I think it would have lowered the stress levels a bit if you had decreased the scope a bit” (S34). Other students felt that the lack of adaptation made it feel demanding: “… somehow you have to manage the workload ... yeah, and it was, you know, it was doable, but it was brutal” (S10). Most of the teaching staff reported that the scope was the same as before the pandemic: “Yes, they do the same assessment as before, the same one. And the exam has stayed the same, too” (TS18).

#### Lack of free practice time

The main category “lack of free practice time” was only assessed for the students; some students were worried about the lack of time and did not feel secure enough ahead of the exam: “Due to the lack of practice time, I didn’t feel confident in the exam at all” (S10). Other students, in turn, reported that they did have enough time: “The free practice times, too [...] and I think everyone has enough time to practise, too” (S12).

#### Questioning personal responsibility

The main category “questioning personal responsibility” described the staff perspective on students studying under this specific regulation. The presentation of theoretical content made available online and usage of theoretical content in the practical courses were difficult for some students as a statement of a teaching staff demonstrated: “You get the impression that those who do not bring a high degree of personal responsibility to the task get left behind. They don’t get carried along any more” (TS03).

#### Course learning gain

The gain from the course was assessed on a scale of less, equivalent, and more. Most of the students and teaching staff stated that gain was equivalent to the semester before. One teaching staff described the gain as follow: “There is no difference; so the work was not qualitatively worse or qualitatively less” (TS04). Having small student groups in the practical courses in particular led to a feeling that students gained more from the courses as stated from the perspective of teaching staff: “By reducing the size of the groups, some students have learned more, or the groups have learned more, because they’re supervised more closely by us, too” (TS07).

#### Support from teachers (students only)

The main category “support from teaching staff” described how students perceived support. Most of the students were very happy about the intensive support: “I know that without coronavirus we would have had much less supervision. And I appreciate that very much, too” (S18). The students described the teaching staff as very helpful. Some students wished the teaching staff had acted more as a team when it came to communications: “Sometimes you had the feeling that it hadn’t quite all been agreed among the team. So you went to one assistant lecturer and you were supposed to do it this way, then you went to the next one and you were supposed to do it differently” (S22).

#### Concern about low quality (separate at the end as has no subcategories)

Students and teaching staff were asked whether they have concerns about lower quality due to the specific regulations in the practical courses. The statements from the perpective of teaching staff as well students showed no concerns regarding lower quality within the practical courses: “So I can’t say that the quality of the teaching is somehow worse or that the information transfer is worse” (S23).

## Discussion

The Covid-19 pandemic posed great challenges for university teaching and dental teaching in particular. Internationally, teaching went completely digital in many cases [5]. Just a few locations reported holding practical courses during the pandemic [5, 11, 17]. Implementation of these courses under the strict social distancing and hygiene regulations showed that students and lectures experienced it differently. The qualitative study conducted in Kiel was able to observe interesting aspects of the ways that students and lecturers experienced this situation.

In their overarching response to being informed that permission to conduct the practical courses had been granted, students and lecturers alike mostly reacted with a sense of relief and hope: on the one hand, it provided the opportunity for practical work, as well as the hope that studies would not be prolonged. Moreover, for the students and their professional future no break in continuity of their practical courses have been observed at Kiel. They could further development their practical skills due to the specific regulations as supplied before and as desired. This desire for practical training was also described in a New Zealand study. Here, students and lecturers were confronted with the pandemic mid-semester due to the earlier start of the semester within the year. The majority of students and lecturers were in favour of continuing their courses, since having less practical training was seen as a negative effect [17]. This same situation was observed in a Jordanian study; however, here the suspension of clinical courses was initiated by the faculty during the semester in progress. This decision was supported by the majority of the teaching staff. However, slightly more than 50% of them, as well as about 50% of students surveyed, saw a negative effect on clinical competence [11].

It was especially in the context of a dental semester conducted entirely digitally that this uncertainty with regard to lengthening the study programme was reported [18–20]. Although the majority of students and lecturers at Kiel expressed relief about courses being conducted, an inner conflict was repeatedly described between feelings of relief and the risks of the necessary treatment of a number of older patients.

There was also uncertainty on the part of the students as to whether the courses would be able to be completed in the given time frames since, although the daily structure was changed, the practical courses were not all extended in length. This was also reflected in comments on the strict unfamiliar entry and exit regulations for students. As our results, demonstrated students perceived it as burdensome and stressful, especially at the beginning of the practical courses. This stress and associated uncertainty was also reported in the New Zealand study [17].

Student compliance with the guidelines was, however, described positively by the lecturers. They tended to comment on the additional time required to organise and monitor the guidelines and regretted that there was less interaction between students and lecturers. This was an effect of the measures that was also observed by the Loch et al. working group [17].

On the one hand students and teaching staff felt relief when starting the pratical courses. However, on the other hand some of the intervieweed students and lectures felt uncertainty due to the possible risk of infection for themselves, as well as the risk of infecting others like patients as described aslo in other studies [11, 17].

The extent to which the necessary measures had an influence on the scope and thus also on the learning gain was described differently. In the Kiel dental clinic, the range of activities to be performed was changed only slightly or not at all. This was possible because by bringing theoretical content forward in time in combination with digital teaching, downtime could be partially compensated for, allowing for more flexibility. This slight reduction was viewed positively by the majority of students and teachers, despite the stress described above. The reduced group size, in particular, and the resulting closer supervision were perceived as positive. Thus, as observed in other studies, there was no negative impact on clinical performance and competency acquisition [11, 17]. Where teaching was exclusively digital, however, this effect was, for understandable reasons, indeed observed in other countries [8, 20, 21].

### Limitations and strengths

Our results are based on the subjective statements of the students and teachers. No generalisations can therefore be drawn from them. However, this is not the intention of a qualitative exploration. The desired participant numbers were nevertheless achieved for both groups and thus the desired saturation was able to be achieved.

Since participation was voluntary, it must be assumed that it was the more committed individuals who took part. This positive selection bias should be taken into account when interpreting the results.

The study’s quality was ensured through consistent adherence to pre-determined quality standards. All interviews were conducted by the same two people using the same interview guide for all students under the same overall conditions. In addition to this, the proceedings from each interview were documented according to a previously agreed protocol.

Consistent adherence to pre-established standards ensured the quality of the study, thus fulfilling the quality criteria for qualitative research [13]. These standards included that all interviews were conducted by the same individuals using the same interview guide for students and lecturers under the same conditions and that the proceedings from each interview were documented according to a previously agreed protocol. However, no inter-coder agreement was cacluated.

## Conclusions

In summary, it became apparent that conducting the practical courses under special coronavirus conditions presented students and lecturers with a variety of new and unfamiliar challenges. The structural and content-related course adjustments required a high degree of flexibility from students and lecturers alike. In actual fact, however, thanks to the modifications made the courses all ran successfully and there were few to no deficits in learning gains. The success of the concept’s implementation – a very demanding process for all involved – was also shown in the avoidance of possible positive coronavirus cases. No one tested positive for coronavirus in the dental clinic during the entire summer semester.

## Supplementary Information

**Additional file 1: Supplementary Table 1**. Consolidated criteria for reporting qualitative studies (COREQ). 32-item checklist.

**Additional file 2.** Interview guide.

## Data Availability

All data generated or analysed during this study are included in this published article.
