# Supplementary material for: Covid-19: teaching and learning in practical courses under special regulations – a qualitative study of dental students and teachers
Source: BMC Med Educ. 2022 Aug 3;22:596. doi: 10.1186/s12909-022-03656-5 (PMC9347151; doi:10.1186/s12909-022-03656-5)
Supplement: Supplementary file 1 — Additional file 1: Supplementary Table 1. Consolidated criteria for reporting qualitative studies (COREQ). 32-item checklist. [file 12909_2022_3656_MOESM1_ESM.docx]

**Supplementary table 1**

Consolidated criteria for reporting qualitative studies (COREQ). 32-item checklist

| **No Item** | **Guide questions/descriptions** | **Page No/**  **comment** |
| --- | --- | --- |
| **Domain 1: Research team an reflexivity** | | |
| Personal characteristics | | |
| 1. Interviewer/facilitator | Which author/s conducted the interview for focus group? | 3 |
| 1. Credentials | What were the researcher’s credentials? *e.g. PHD, MD* | Title page  5 |
| 1. Occupation | What was their occupation at the time of the study? | Title page,  5 |
| 1. Gender | Was the researcher male or female? | 3 |
| 1. Experience and training | What experience or training did the researcher have? | 5 |
| Relationship with participants | | |
| 1. Relationship established | Was a relationship established prior to study commencement? | no |
| 1. Participants knowledge of the interviewer | What did the participants know about the researcher? *e.g. personal goals, reasons for doing the research* | Professional  background |
| 1. Interviewer characteristics | What characteristics were reported about the interviewer/facilitator? *e.g. Bias, assumptions, reasons and interests in the research topic* | 10-11 |
| **Domain 2: study design** | | |
| Theoretical framework | | |
| 1. Methodological orientation and Theory | What methodological orientation was stated to underpin the study? *e.g. grounded theory, discourse analysis, ethnography, phenomenology, content analysis* | 4 |
| Participant selection | | |
| 1. Sampling | How were participants selected? *e.g. purposive, convenience, consecutive, snowball* | 4 |
| 1. Method of approach | How were participants approached? *e.g. face-to-face, telephone, mail, email* | 3 |
| 1. Sample size | How many participants were in the study? | 4 |
| 1. Non-participant | How many people refused to participate or dropped out? Reasons? | 5 |
| Setting | | |
| 1. Setting of data collection | Where was the data collected? *e.g. home, clinic, workplace* | 3-4 |
| 1. Presence of non-participants | Was anyone else present besides the participants and researcher? | no |
| 1. Description of sample | What are the important characteristics of the sample? *e.g. demographic data, data* | 5 |
| Data collection |  |  |
| 1. Interview guide | Were questions, prompts, guides provided by the authors? Was it pilot tested? | 4 |
| 1. Repeat interviews | Were repeat interviews carried out? If yes, how many? | no |
| 1. Audio/visual recording | Did the research use audio or visual recording to collect the data? | 4 |
| 1. Field notes | Were field notes made during and/or after the interview of focus group? | yes |
| 1. Duration | What was the duration of the interview or focus group? | 5 |
| 1. Data saturation | Was data saturation discussed? | 10 |
| 1. Transcripts returned | Were transcripts returned to participants for comment and/or correction? | no |
| **Domain 3: analysis and findings** | | |
| Data analysis | | |
| 1. Number of data coders | How many data coders coded the data? | 5 |
| 1. Description of the coding tree | Did authors provide a description of the coding tree? | 4-5 |
| 1. Derivation of themes | Were themes identified in advance or derived from the data? | 5-9 |
| 1. Software | What software, if applicable, was used to manage the data? | 4 |
| 1. Participant checking | Did participants provide feedback on the findings? | no |
| Reporting | | |
| 1. Quotations presented | Were participant quotations presented to illustrate the themes/findings? Was each quotation identified? *e.g. participants number* | 5-9 |
| 1. Data and findings consistent | Were there consistency between data presented and the findings? | yes, 5-9 |
| 1. Clarity of major themes | Were major themes clearly presented in the findings? | 5-9 |
| 1. Clarity of minor themes | Is there a description of diverse cases or discussion of minor themes? | 5-9 |
