# Supplementary material for: Covid-19: teaching and learning in practical courses under special regulations – a qualitative study of dental students and teachers
Source: BMC Med Educ. 2022 Aug 3;22:596. doi: 10.1186/s12909-022-03656-5 (PMC9347151; doi:10.1186/s12909-022-03656-5)
Supplement: Supplementary file 2 — Additional file 2. Interview guide. [file 12909_2022_3656_MOESM2_ESM.pdf]

Interview guide

Topic: Implementation of specific regulations in the practical dental courses

- How did you find out about the implementation of the practical dental courses?
- What immediately went through your head?
- How did you perceive the information?
- In your opinion, how did the dental clinic / teaching staff react to the situation?

Topic: Adaptation of the course structure due to specific regulations

The information then came that the courses should be carried out under specific regulation:

- How did you get this information?
- What did you spontaneously think about?
- How do you see this form of practical course in terms of examinations?
- How do you rate the changed structure of the courses?
- Do you have the impression that you have learned more, less or the same amount?
- How do you rate this?
